# Can Molecular Biomarkers Help Reduce the Overtreatment of DCIS?

**DOI:** 10.3390/curroncol30060433

**Published:** 2023-06-13

**Authors:** Ezra Hahn, Danielle Rodin, Rinku Sutradhar, Sharon Nofech-Mozes, Sabina Trebinjac, Lawrence Frank Paszat, Eileen Rakovitch

**Affiliations:** 1 Radiation Medicine Program, Princess Margaret Cancer Centre, Toronto, ON M5G 2C4, Canada; 2 Department of Radiation Oncology, University of Toronto, Toronto, ON M5T 1P5, Canada; 3 Institute for Clinical Evaluative Sciences, Toronto, ON M4N 3M5, Canada; 4 Dalla Lana School of Public Health, University of Toronto, Toronto, ON M5T 3M7, Canada; 5 Department of Laboratory Medicine and Pathobiology, University of Toronto, Toronto, ON M5S 1A8, Canada; 6 Department of Pathology, Sunnybrook Health Sciences Centre, Toronto, ON M4N 3M5, Canada; 7 Department of Radiation Oncology, Sunnybrook Health Sciences Centre, Toronto, ON M4N 3M5, Canada

**Keywords:** DCIS, ductal carcinoma in situ, biomarkers

## Abstract

Ductal carcinoma in situ (DCIS), especially in the era of mammographic screening, is a commonly diagnosed breast tumor. Despite the low breast cancer mortality risk, management with breast conserving surgery (BCS) and radiotherapy (RT) is the prevailing treatment approach in order to reduce the risk of local recurrence (LR), including invasive LR, which carries a subsequent risk of breast cancer mortality. However, reliable and accurate individual risk prediction remains elusive and RT continues to be standardly recommended for most women with DCIS. Three molecular biomarkers have been studied to better estimate LR risk after BCS—Oncotype DX DCIS score, DCISionRT Decision Score and its associated Residual Risk subtypes, and Oncotype 21-gene Recurrence Score. All these molecular biomarkers represent important efforts towards improving predicted risk of LR after BCS. To prove clinical utility, these biomarkers require careful predictive modeling with calibration and external validation, and evidence of benefit to patients; on this front, further research is needed. Most trials do not incorporate molecular biomarkers in evaluating de-escalation of therapy for DCIS; however, one—the Prospective Evaluation of Breast-Conserving Surgery Alone in Low-Risk DCIS (ELISA) trial—incorporates the Oncotype DX DCIS score in defining a low-risk population and is an important next step in this line of research.

## 1. Introduction

There has been a rise in DCIS diagnosis with population screening with mammography, such that DCIS now represents more than one in every four cases of newly diagnosed breast cancers [1,2]. Approximately 54,000 women in the US and 5000 in Canada are diagnosed annually with DCIS [3], and treatment is recommended [4] as part of a standard of care to reduce the subsequent risk of developing invasive breast cancer (IBC) or DCIS in the same breast, referred to as local recurrence (LR) [5,6]. Breast conserving surgery (BCS) is the modality of treatment for the majority of women with DCIS, which results in 10-year risks of LR ranging from 12–48% [7,8]. Half of LRs after BCS are IBC [7,9] necessitating additional treatment including breast surgery (BCS or mastectomy) with axillary lymph node sampling, anti-estrogen hormonal therapy for many, chemotherapy (if the LR is invasive and indicated), and/or radiation therapy (RT), in an effort to reduce the subsequent risk of breast cancer mortality [9,10,11]. Our current inability to identity the subset of individuals with DCIS at risk of developing IBC culminates in recommendations that all women with DCIS undergo treatment.

Most women diagnosed with DCIS are treated with BCS followed by the administration of post-operative breast radiotherapy (RT). The Early Breast Cancer Trialists’ Collaborative Group [7] performed a meta-analysis of randomized control trials evaluating the addition of post-operative breast radiotherapy (RT) after breast-conserving surgery (BCS); the four trials included in the meta-analysis were the NSABP B17 [12], EORTC 10853 [13], UK DCIS trial [14], and Swedish DCIS (SweDCIS) trial [15]. The meta-analysis included 3792 women and found RT to be beneficial in reducing the risk of local recurrence in all subgroups of women (defined by clinical or pathological features) with DCIS [13,15,16,17]. The 10-year cumulative incidence of LR was 28.1% for women treated by BCS alone and 12.9% for women treated with adjuvant radiation (*p* < 0.00001) (Hazard ratio = 0.46, (SE) 0.05, 2 *p* < 0.00001) [7]. More contemporary data also show the benefit of adjuvant RT in reducing the risk of local recurrence after BCS for DCIS [11,18]. The reduction in local recurrence risk, importantly, also includes reduction in recurrence as an invasive breast cancer, referred to as an invasive local recurrence. Such recurrences are psychologically distressing and require further treatment that often includes further surgery, including mastectomy, and chemotherapy if indicated, due to the associated increased risk of breast cancer mortality [9,11]. Adjuvant RT has also been shown to result in lower rates of mastectomies. While some local recurrences can be managed with another breast conserving surgery, data from randomized trials and observational population-based studies report that mastectomy is the most common treatment after local recurrence. In a population-based analysis based in Ontario, Canada, receipt of adjuvant RT was associated with a higher 10-year mastectomy free survival (87.3%) as compared to treatment with BCS alone (82.7%) (*p* < 0.01), with an associated decreased hazard ratio for mastectomy of 0.71 (95% CI: 0.60, 0.84, *p* < 0.0001) [18].

Although breast RT is efficacious, it can be associated with significant early effects such as fatigue, skin erythema, and desquamation, late effects such as breast pain, skin telangiectasia, induration, and hyperpigmentation, which can adversely affect cosmesis and quality of life, and rare but serious life-threatening side effects such as second cancers [19] and heart disease [20,21]. In addition, breast RT is costly to deliver for the healthcare system and poses a risk of financial toxicity for patients and their families [22,23]. Most women with DCIS are generally healthy, and have a low risk of breast cancer related mortality. Additionally, although RT results in decreased rates of local recurrence, it is unlikely to improve overall survival. Therefore, provided a group of women with low risk of LR after BCS could be identified, there is great interest in de-escalation of therapy through the omission of RT [7,24,25,26].

## 2. Clinicopathological Factors to Identify Cases at Low Risk of Local Recurrence after BCS Where the Omission of RT Can Be Considered

Several clinical trials have examined the impact of the omission of breast RT in patients with low-risk clinical and pathological features of DCIS including age at diagnosis > 45 or 50 years [10,17,27,28,29], tumor size ≤ 2.5 cm, nuclear grade 1 or 2 [30,31], and negative resection margins (Table 1) [10,30,32,33,34,35]. The Eastern Cooperative Oncology Group-ACRIN E5194 study is a prospective single-arm study that evaluated the omission of breast RT in a subset of women with ”low risk” DCIS. To be considered low risk, eligibility criteria included: low/intermediate-grade disease with tumor size ≤ 2.5 cm (cohort 1, *N* = 561), or high-grade DCIS with tumor size ≤ 1 cm (cohort 2, *N* = 104). Margins were all negative with a minimum width of 3 mm. In this study, the risk of LR at 12 years was 14.4% and 24.6% in cohorts 1 and 2, respectively [36]. Wong et al. reported on another prospective wide local excision alone cohort and included 158 women with grade 1 or 2 DCIS with resection margins ≥ 10 mm, and found a 10-year risk of LR of 15.6% [37]. The Radiation Therapy Oncology Group (RTOG) 9804 study [38] randomized 636 women with low-risk DCIS treated by BCS to whole breast RT versus observation. For eligibility, nuclear grade needed to be grade 1 or 2, and tumor size ≤ 2.5 cm, with clear margins. The mean DCIS tumor size was 0.6 cm. The 15-year risk of LR was 15.1% in those treated with BCS alone, with a risk of invasive LR of 9.5% [39]. 

A population-based study of women with pure DCIS and low-risk disease based on clinicopathologic features, who were selected for BCS alone, identified 1867 women between 1994–2003 [33]. The 10-year risk of LR and invasive LR, with a median follow up of 10.1 years, was 20.0% and 10.0%, respectively. Within the population cohort, there were 741 individuals with all the following features: age > 50 years, unifocal disease, low to intermediate grade, and margin-negative resection; the 10-year rate of LR in this subgroup was 14.4% following treatment by BCS alone corroborating findings from prospective studies [33]. The UK National Health Service Breast Screening Programme identified a cohort of 5497 patients who were treated by BCS alone for DCIS [11] and noted a 7.2% rate of local recurrence with a median follow up of 62 [11]. Overall, randomized and nonrandomized studies report that among women with selected low-risk features of DCIS, treatment by BCS alone is associated with 10-year risks of LR ranging from 11–14%. As a result of past studies, treatment guidelines for women with low-risk DCIS based on clinicopathologic features include consideration of omission of breast RT [40].

However, despite efforts of past studies and guideline recommendations, population-based patterns of care analyses from the United States and Canada report that most women diagnosed with DCIS are treated with adjuvant radiation after BCS [41,42,43]. A SEER-based study from 1991–2010 of 121,080 individuals diagnosed with DCIS in the United States, showed that 67.1% of women treated with BCS received adjuvant RT. Moreover, the rates of BCS alone without RT declined from 29.8% to 22.3% with a corresponding rise in the use of post-operative adjuvant breast RT (*p* > 0.001). Among patients with low-grade DCIS, 52% received RT; similarly, 61% of patients with intermediate-grade DCIS received RT [41]. An analysis from the National Cancer Database from 2002–2013 of 66,079 patients diagnosed with DCIS found that of patients treated with BCS, 71% received adjuvant RT [43]. A population-based analysis of women with screen-detected DCIS diagnosed through the Ontario Breast Screening program from 1991–2000 found that in the year 2000, 58% of women with pure DCIS treated with BCS received RT [42]. The continued use of RT reflects clinicians’ and patient concerns that even among the lowest risk DCIS based on clinicopathologic features, LR risk estimates of 11–14% at 10 years [36,44] is deemed not sufficiently low to omit breast RT.

## 3. Adjuvant Endocrine Therapy

The NSABP (National Surgical Adjuvant Breast and Bowel Project) B-24 study evaluated the effect of five years of adjuvant tamoxifen in patients with DCIS treated with lumpectomy and radiation [45,46]; a subsequent analysis of 732 cases with complete hormone receptor information, report estrogen receptor (ER) was positive in 76% of patients. In this subset of women with ER+ DCIS, treatment with tamoxifen (versus placebo) significantly decreased the risk of LR at 10 years (HR, 0.64; *p* = 0.003). Overall, the administration of five years of adjuvant endocrine therapy was associated with a small, absolute reduction in the risk of recurrent DCIS but no significant reduction in the risk of IBC (HR 0.79; 95% CI 0.62 to 1.01) or mortality (RR 1.11; 95% CI 0.89 to 1.39) [47]. The use of endocrine therapy in the management of DCIS is inconsistent, which may be related to its lack of survival benefit and associated adverse effects [48].

## 4. Can Molecular Biomarkers Help De-Escalate the Treatment of DCIS?

To have clinical utility, a biomarker assay should improve risk stratification by identifying women with a sufficiently low risk of local recurrence, such that the absolute benefit from RT would be very small. Alternatively, a biomarker predictive of the befit of RT would identify those patients for whom RT would be expected to yield the largest reduction in risk of recurrence through identifying patients with a high risk of local recurrence after BCS. Finally, implementation of such biomarkers should be feasible to attain in the clinical workflow without onerous complexity, such as a report that is clinician and patient facing that can be used in clinical decision making. To date, three molecular assays have been clinically evaluated in DCIS (Table 2) [49,50,51,52].

### 4.1. Oncotype DX DCIS Score (Exact Sciences)

The Oncotype DX *DCIS score* (DS) is one of the first molecular expression assays evaluated in DCIS. The DS comprised 12 of the 21 genes of the Oncotype DX Recurrence score [54]; there are seven genes related to cancer (Ki67, STK15, Survivin, Cyclin B1, MYBL2, progesterone receptor, and GSTM1) along with five genes for reference (ACTB (beta-actin), GAPDH, RPLPO, GUS, and TFRC). The DS is a numeric value ranging from 0–100. Patients are categorized into three risk groups based on different cut offs of the DS, defined as: low (<39), intermediate (39–54), and high risk (≥55). An analysis from the E5194 study demonstrated the DS as an independent predictor of LR after treatment with BCS alone (hazard ratio (HR) = 2.31, 95% confidence interval [CI]:1.15 to 4.49; *p* = 0.02) [49]. The 10-year LR and invasive LR rates after treatment by BCS were 10.6%, 20.7%, and 25.9% (*p* = 0.006) and 3.7%, 12.3%, and 19.2% (*p* = 0.003) for individuals with low-, intermediate-, and high-risk DS scores, respectively. 

The DS assay was externally validated in a retrospective, population-based cohort analysis of 571 women diagnosed with DCIS in Ontario treated by BCS alone [51]. Using multivariate analysis, the DS was significantly associated with the risk of LR (HR = 1.68, 95% CI: 1.08, 2.62 *p* = 0.02). The 10-year rates of LR after treatment with BCS alone were 12.7% for the low-risk group, and 33% and 28% for intermediate- and high-risk DS categories, respectively (*p* ≤ 0.001 for the 3-way comparison). In addition, multifocal DCIS (HR = 1.97; 95% CI: 1.27, 3.02), tumour size > 10 mm (HR = 2.07; 95% CI: 1.15, 3.83) and age at diagnosis (HR = 1.75; 95% CI: 1.07, 2.76) were also shown to be additional independent predictors of LR.

### 4.2. DCISionRT Decision Score (PreludeDx)

The DCISionRT Decision Score (PreludeDx) is an assay comprised of six genes evaluated by immunohistochemistry (Her2, Ki-67, Cox-2, SIAH2, FOXA1, p16) and four clinicopathological factors (age at diagnosis, tumor size, palpability of DCIS lesion, margin status) [49,50,51,52,55] scaled from 0–10 to produce the Decision Score. The low-risk group was defined as individuals with a Decision Score ≤ 3, selected to identify a group with a 10-year LR risk < 10% and a 10-year ICB risk ≤ 6% [52]. The Decision Score was initially reported to be associated with LR risk and RT benefit in a retrospective analysis of 526 individuals diagnosed with DCIS from two institutions in Sweden and the University of Massachusetts [52] treated with BCS +/− breast RT. Among 196 individuals with a low-risk Decision Score, the 10-year LR risk was 8% for those treated by BCS alone and 7% for those treated with BCS + RT. For 278 individuals in the elevated risk group, the 10-year LR risk was 23% for those treated by BCS alone and 11% for those treated with BCS + RT. 

Another retrospective study evaluated the Decision Score in 455 patients treated in the Kaiser Permanente Northwest cohort (78 patients were treated by BCS alone and 377 received RT) [56]. Median follow up was 10.4 years and 24% of patients were prescribed endocrine therapy for an average of 4.2 years. For those in the low-risk group, the 10-year LR risk was 10% for those treated by BCS alone and 5% for those treated with BCS and adjuvant RT, while for those with an elevated risk group, the 10-year LR risk was 30% for those treated by BCS alone and 10% for those treated with BCS and adjuvant RT. Among patients in the elevated risk group treated by BCS alone, the 10-year risk of invasive LR was 21%, but reduced to 12% when excluding woman with positive margins and assessing patients with clear resection margins only.

An external validation study using data from the SweDCIS randomized trial [57] using the predefined threshold of 3.0 to define low- and elevated-risk groups, reported the assay was not predictive of RT benefit nor was it prognostic for LR (*p*-value = 0.24 for the interaction of the RT effect and Decision Score (<3 versus >3) [57]). Analyses examining interactions of assay with multiple thresholds ranging from 1.0–3.0 with the effect of RT were subsequently performed. The investigators found a statistically significant interaction with RT using a DS threshold of 2.8 for the development of invasive LR but not with total breast events (LR) [57]. 

A subsequent study evaluated a modified Decision Score combining the initial Decision Score signature with EGFR/HER2/KRAS expression, termed the Residual Risk (RRt) subtype, to define three new categorical risk groups: “(1) Low Risk group (DS ≤ 2.8 without RRt), (2) Elevated Risk group (DS > 2.8 without RRt), or (3) Residual Risk group (DS > 2.8 with RRt)”. The assay was evaluated in 926 individuals from the original US (UMass and Kaiser Permanente) and Swedish (UUH) cohorts with additional patients from the Royal Melbourne Hospital and Royal Women’s Hospital in Australia. The 10-year risk of LR in the low-risk group did not change significantly with RT, with an overall risk of 5.1%. In the elevated risk group and residual risk group, the 10-year risk of LR following BCS alone was 21% and 42% and was 4.9% and 14.7% following treatment with BCS + RT, respectively [53].

### 4.3. Oncotype 21-Gene Recurrence Score (Exact Sciences)

**Figure 1:**
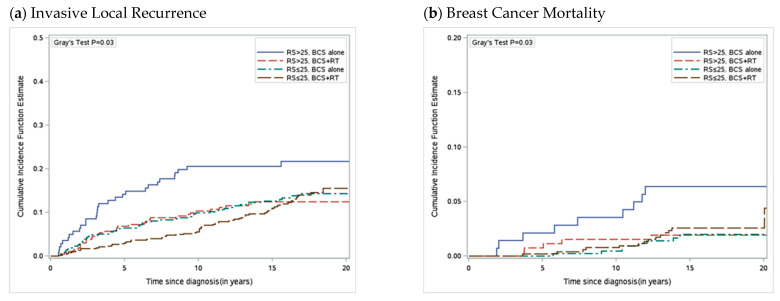
Cumulative Incidence of Invasive Local Recurrence and Breast Cancer Mortality by Recurrence Score and Treatment. (**a**) Cumulative incidence function for development of invasive local recurrence as a first event with competing risks of ipsilateral DCIS LR, contralateral breast cancer and death, (**b**) breast cancer mortality with competing risk of death from other causes. DCIS = ductal carcinoma in situ; LR = local recurrence.

The 21-Gene Oncotype DX Breast Recurrence Score (RS) is a validated biomarker currently used in routine clinical practice, associated with the risk of developing metastases and chemotherapy benefit in patients with early, invasive breast cancer [58,59]. This biomarker started being incorporated into society guidelines in 2007 with increasing adoption over the following years. The 21-gene RS was also shown to be prognostic in individuals with DCIS where one-third of DCIS tumors had a high RS (>25). Among women with primary DCIS tumors that had a high RS, there was an associated higher cumulative risk of invasive local recurrence and associated increased risk of breast cancer death (Figure 1). In woman aged ≤50 treated with BCS alone, a high RS was associated with a hazard of breast cancer death of greater than 11 (HR = 11.27, 95% CI = 3.00 to 42.33, *p* < 0.001), with a 20-year risk of breast cancer death of 9.4% (95% CI = 2.3 to 22.5). Additionally, among women with a high RS, receipt of adjuvant RT was associated with a relative reduction of 71% (HR = 0.29, 95% CI = 0.10 to 0.89, *p* = 0.03), and absolute reduction of 5%, in the 20-year cumulative risk of breast cancer related death [60]. Among women with a low RS, most deaths were due to other nonbreast cancer related causes, and the risk of death from breast cancer was low.

Significant progress has been made in the discovery and evaluation of molecular assays to help improve risk estimates for individual patients with DCIS. However, prospective validation of the novel biomarker assays in DCIS is needed to ensure the biomarker assays provide accurate, reproducible estimates of recurrence risks in independent populations treated with BCS for DCIS. Given the current lack of prospective validation, it is challenging to interpret biomarker study results generalizing various scores and associated risks outside of the study in which it was conducted and internal validation reported. Treatment guidelines, therefore, do not currently recommend the routine use of molecular assays in the management of DCIS.

## 5. Predictive Modeling

Precision oncology is an emerging approach for cancer treatment that aims to recognize the heterogeneity of cancer in order to individualize cancer risk assessments and treatment recommendations. A key feature of precision medicine is the ability to accurately estimate an individual patient’s risk of developing recurrent disease because prognostic estimates can be used to inform clinicians and patients of the likelihood of an outcome, which can then be used as a guide for treatment decision making. To accomplish this, models that reflect associations between a baseline feature (clinical, pathological, or molecular) in the initial data must then be proven to also accurately predict outcomes when applied to comparable, independent populations in other settings, known as validation. There has been extensive proliferation of molecular biomarkers and predictive models in healthcare, yet meaningful clinical impact from these assays and models has been limited. To have clinical utility, it must be shown that integration of a novel-molecular biomarker in a predictive model can more accurately estimate outcomes compared to estimates based on routine clinicopathologic features on their own. That is, the use of a novel biomarker or molecular assay must improve our predictive power beyond readily available clinicopathological factors, and that the benefit is of a magnitude that reaches clinical impact and ideally is cost effective. Such judgements are both clinical and statistical. Selection of variables to put in the model are based on prior research, clinical reasoning, and statistical methods. Predictive models need to be created thoughtfully, with appropriate sample size, relevant input variables with systematic homogeneity of measurement, accurate outcomes data with sufficient follow up and validation.

Validation of a predictive model is essential because a lack of critical evaluation of prognostic or predictive models can result in poorly fit models, which can result in inaccurate predicted outcomes for new subjects precluding generalizability to clinical relevance. External validation is a key step in which a predictive model is tested on data it was not created from. Measurement of the predictive accuracy of a model is performed by calibration and discrimination—that is to give an accurate estimate of the model’s ability to accurately estimate the risk of an event for a given individual. Calibration is the assessment of the degree to which the predicted risk matches the observed risk. For example, regarding LR in DCIS, calibration plots can compare the model-based predicted 10-year risks of LR against the observed 10-year risks of LR derived from Kaplan–Meier estimates and then examine how closely aligned the estimated risks are to the observed risks. Poorly calibrated models are not useful and are potentially risky, providing inaccurate estimates of risk if used to guide decision making in a clinical setting. Discrimination can be measured in multiple ways, perhaps most commonly the concordance (c) statistic, which measures the area under the receiver operating curve (AUC). The c statistic is an estimate of the probability of concordance between predicted and observed estimates. A c statistic of 0.5 indicates no discrimination, while a value of 1.0 indicates perfect discrimination. Given the many important elements in a high-quality predictive model, guidelines exist to aid in standardization of development and validation of models as in the transparent reporting of a multivariable prediction model for individual prognosis or diagnosis (TRIPOD).

With regard to the management of DCIS, predictive models are needed to accurately predict an individual’s risk of developing LR and, more importantly, invasive LR following treatment with BCS. This information can then be used to make individualized recommendations weighing the risks of recurrence against the risks and benefits of treatment, including breast RT. Two predictive models in DCIS have been reported [8,55]. One was based on a collaborative analysis combining data from the ECOG ACRIN E5194 and Ontario cohort DCIS studies, and compared three models in terms of their performance in predicting the 10-year risk of LR after treatment by BCS alone (all with clear margins); one model included the 12-gene DCIS Score alone, another was based on tumor size, age at diagnosis, and year of diagnosis, and lastly a model that combined all three and adjusted for year of diagnosis. The best performing model incorporated tumor size, age at diagnosis and year of diagnosis, as well as the 12-gene DCIS Score. The model incorporating DS and clinicopathologic factors had superior discrimination to models without the DS, with c statistics of 0.7 versus 0.68, respectively. Notably, integrating the 12-gene DCIS Score with tumor size and age at diagnosis identified a subgroup of patients with an estimated 10-year risk of LR < 10%; the predicted 10-year risk of LR for a woman age ≥ 50 years, with tumor size ≤ 2.5 cm, nuclear grade 1 or 2, clear margins without multifocality and a low-risk (<39) 12-gene DCIS Score was 6.8% (range: 4.4–9.5%) [8]. Based on this work, the DCIS Score report was revised to provide 10-year risk of LR after BCS based on the DCIS Score, tumor size, and age at diagnosis. Another predictive modeling analysis explored the effect of all clinical and pathological factor (CPF) information alone, CPF with estrogen receptor (ER) and HER2 expression, and CPF and the DCIS score. Internal validation was performed by bootstrapping and accuracy of the models was assessed by c statistics along with calibration plots. Incorporation of CPF and the DCIS Score resulted in the strongest prediction model. Both models found that integrating the 12-gene DCIS Score with clinicopathological features of DCIS improved the predicted estimates of 10-year LR risk following BCS for DCIS and was particularly better at predicting low (≤10%) individual 10-year LR risks after treatment with BCS alone (Table 3) [55]. 

## 6. Future Clinical Trials

Future clinical trials are studying how to best identify a subgroup of women who have low-risk DCIS and who can have de-escalation in therapy. Four prospective trials are designed to follow patients with presumably low-risk DCIS diagnosed on core biopsy and omit surgical resection (LORIS, COMET LORD, and LORETTA). The four trials, opened in different parts of the world, vary slightly in their inclusion criteria. The Comparing an Operation to Monitoring, With or Without Endocrine Therapy (COMET) Trial For Low Risk DCIS trial (ClinicalTrials.gov accessed on 1 April 2023. Identifier: NCT02926911) hypothesizes that an active monitoring approach for patients with low-risk DCIS does not have inferior outcomes to BCS [24]. In this randomized study, the control arm is surgery +/− RT with an experimental arm of active monitoring; use of endocrine therapy in both arms is optional. The primary outcome is invasive LR. The LORD (Low Risk DCIS) (ClinicalTrials.gov accessed on 1 April 2023. Identifier: NCT02492607) study is a nonrandomized, international, phase III noninferiority trial. Patient preference determines arm allocation, which include active surveillance or standard treatment according to local policy; standard treatment could be BCS plus or minus RT, or mastectomy, and possibly followed by hormonal therapy. The primary endpoint is ipsilateral invasive breast tumor-free rate at 10 years. The main aim of the study is to determine the safety of active surveillance for screen-detected low-risk DCIS. The Japan Clinical Oncology Group 1505 trial is a single-arm multicenter prospective study that evaluated treatment with endocrine therapy, without surgery, for patients with low-grade ductal carcinoma in situ. Eligibility criteria included low to intermediate nuclear grade DCIS without comedo necrosis, and high estrogen receptor positivity defined by immunohistochemical staining. A study from Japan looked at the upstage rates among 152 women who were diagnosed with DCIS on core and would have met the inclusion criteria for one of the active surveillance trials [61]. Invasive carcinoma was identified in 12–25% of the patients on excision. Most of the occult invasive cancers were 5 mm or smaller. This study highlights the need to appropriately select patients with a very low risk for active surveillance on appropriately designed clinical trials in order to not enroll patients with invasive disease or aggressive biology. How molecular biomarkers may contribute to optimizing patient selection for possible active surveillance trials remains to be seen, and further work is needed to correlate biomarkers on core biopsy to full surgical specimens on excision.

The aforementioned trials do not use molecular biomarkers to identify subgroups of low-risk DCIS beyond clinicopathologic factors. A prospective cohort study by the Ontario Clinical Oncology Group (OCOG) is called the Prospective Evaluation of Breast-Conserving Surgery Alone in Low-Risk Ductal Carcinoma in Situ (DCIS) (ELISA) trial (ClinicalTrials.gov accessed on 1 April 2023. Identifier: NCT04797299). This prospective study will assess patients with low-risk DCIS defined by both clinicopathological criteria as well as the Oncotype DX DCIS score, and will evaluate if this group of women with very low risk of local recurrence can be treated with breast conserving surgery alone and not require adjuvant radiation. Eligible patients must be >45 years old without microinvasion, tumor size < 2.5 cm, unifocal disease, surgical resection margins clear (≥2 mm or negative re-excision), with a DCIS Score < 39 and a predicted 10-year risk of local recurrence < 10% following BCS alone. The study plans will accrue 526 women (https://clinicaltrials.gov/ct2/show/NCT04797299, accessed on 24 November 2022).

## 7. Conclusions

In summary, much progress has been made with molecular assays in improving risk predictions of woman with DCIS treated with BCS. The ability to accurately identify which women are at a very low risk of recurrence after BCS alone and for whom adjuvant RT could be safely omitted, or to predict which women are at a high risk of recurrence and for whom RT would be beneficial, would allow personalization of care based on individualized risk. Molecular assays improve prediction beyond clinicopathologic features alone; however, further research is needed to examine the improved accuracy of the assays through predictive modeling in addition to external validation of the models to improve risk estimates and estimated benefit of treatment intervention in the management of patients with DCIS.

## Figures and Tables

**Table 1:** Trials Evaluating the Omission of Breast RT in DCIS.

Study	Design	*N*	Eligibility	12 Year LR Risk	12 Year Invasive LR Risk
ECOG-ACRIN 5194	Prospective single arm cohort study of BCS alone	627	Cohort 1 (≤2.5 cm, grade 1 or 2) Cohort 2 (≤1 cm, grade 3) Resection margins > 3 mm	Cohort 1: 14.4% Cohort 2: 24.6%	Cohort 1: 7.4% Cohort 2: 13.4%
Boston Cohort	Prospective single arm cohort study of BCS alone	158	Low/intermediate grade DCIS Margins > 1 cm	15.6%	
RTOG/NRG 9804	Randomized clinical trial	636	Tumor size < 2.5 cm Nuclear grade 1 or 2 Margins > 3 mm 60% received tamoxifen	15-yr risk: BCS alone: 15.1% BCS + RT: 7.1%	BCS alone: 9.5% BCS + RT: 5.4%

**Table 2:** Genomic Biomarkers in DCIS.

	Genes	Risk Groups	Multivariable Hazard Ratio (HR)	Comments
Oncotype DCIS Score [49,50,51] (Exact Sciences)	Ki67, STK15, Survivin, cyclin B1, MYBL2, PR, GSTM1 + reference genes (b-actin, GAPDH, RPLPO, GUS, TFRC) *Scaled from 0–10*	Low: <39 Intermediate: 39–54 High: >55	DS/50 = 2.1 (1.4, 3.1) Age < 50 yrs = 1.83 (1.2, 2.7) Tumor size > 10 mm = 1.7 (1.0, 2.9) Multifocality = 1.98 (1.3, 2.9) Pos. margins = 1.5 (1.0, 2.1)	Validated in E5194 and Ontario DCIS cohorts; Integration of DS improves prediction of 10 yr LR risk after BCS compared to clinical factors alone or with ER, PR, HER2 status [8]; Clinical factors contribute to LR risk prediction.
Decision Score [52] (Prelude DX)	Her2, Ki-67, Cox-2, SIAH2, FOXA1, p16, age, tumor size, palpability of DCIS lesion, margin status (DCISionRT) *Scaled from 0–10 to produce the Decision Score*	Low: ≤3 Elevated: >3	HR for effect of RT as a function of the DS: Low Risk DS = 0.7 (0.3–1.6) High Risk DS = 0.3 (0.1–0.5)	RT benefit prediction; Clinicopathologic factors did not maintain significance; Not validated in SweDCIS randomized trial; significant interaction found with RT using threshold of 2.8.
Decision Score and Residual Risk subtype [53] (RRt) (Prelude DX)	Decision Score (threshold 2.8) combined with EGFR/HER2/KRAS expression	Low Risk: (DS ≤ 2.8 without RRt) Elevated Risk: (DS > 2.8 without RRt) Residual Risk: (DS > 2.8 with RRt).	10-yr LR after BCS alone by groups: Low risk = 5.1% Elevated risk = 21% Residual risk = 42%	No benefit with RT in low-risk group; 10 yr LR risk after BCS + RT: Elevated risk = 4.9% Residual risk = 14.7%

**Table 3:** 10-year risks of any Local Recurrence (DCIS or Invasive) after Breast-Conserving Surgery Alone by combinations of age, tumor size, and DCIS Score.

10-Year Risk of Local Recurrence (%) ^a^ (Range ^b^) by DCIS Score Group				
Tumor Size (cm)	Age (yr)	Low DCIS Score (0–38)	Intermediate DCIS Score (39–54)	High DCIS Score (55–100)
≤1	≥50	7.2 (5.3–10.0)	11.3 (10.2–12.7)	14.6 (12.9–23.1)
	<50	10.2 (7.4–13.9)	15.8 (14.1–17.4)	19.6 (17.7–30.7)
1.1–2.5	≥50	10.1 (7.3–12.6)	13.9 (12.8–15.6)	19.5 (15.8–28.7)
	<50	14.5 (10.1–17.2)	18.9 (17.4–21.1)	23.2 (21.4–37.2)
>2.5	≥50	20.4 (14.9–27.0)	29.1 (27.4–33.3)	41.1 (33.8–54.4)
	<50	30.2 (20.6–36.1)	39.5 (36.6–43.6)	48.6 (44.1–66.5)

^a^ Average risk for E5194 and Ontario DCIS Cohort patients in DCIS Score groups. ^b^ Risks at boundaries of DCIS Score groups.
